# Preoperative risk factors associated with left ventricular dysfunction after bariatric surgery

**DOI:** 10.1038/s41598-024-52623-1

**Published:** 2024-01-25

**Authors:** Lisa M. D. Grymyr, Gunnar Mellgren, Adrian McCann, Eva Gerdts, Klaus Meyer, Saied Nadirpour, Johan Fernø, Bjørn G. Nedrebø, Dana Cramariuc

**Affiliations:** 1 Department of Heart Disease, Haukeland University Hospital, Jonas Liesvei 65, 5021 Bergen, Norway; 2 Department of Clinical Science, University of Bergen, Bergen, Norway; 3 Mohn Nutrition Research Laboratory, Department of Clinical Science, University of Bergen, Bergen, Norway; 4 Hormone Laboratory, Department of Medical Biochemistry and Pharmacology, Haukeland University Hospital, Bergen, Norway; 5 Bevital AS, Bergen, Norway; 6 Center for Research on Cardiac Disease in Women, Department of Clinical Science, University of Bergen, Bergen, Norway; 7 Department of Medicine, Haugesund Hospital, Haugesund, Norway

**Keywords:** Obesity, Cardiovascular diseases, Biomarkers, Medical imaging, Risk factors

## Abstract

A large proportion of patients with severe obesity remain with left ventricular (LV) dysfunction after bariatric surgery. We assessed whether preoperative evaluation by echocardiography and inflammatory proteins can identify this high-risk group. In the Bariatric Surgery on the West Coast of Norway study, 75 patients (44 ± 10 years, body mass index [BMI] 41.5 ± 4.7 kg/m^2^) were prospectively evaluated by echocardiography and inflammatory proteins (high-sensitivity C-reactive protein [hsCRP], serum amyloid A [SAA] and calprotectin) before and one year after Roux-en-Y gastric bypass surgery. LV mechanics was assessed by the midwall shortening (MWS) and global longitudinal strain (GLS). Bariatric surgery improved BMI and GLS, and lowered hsCRP, calprotectin and SAA (p < 0.05). MWS remained unchanged and 35% of patients had impaired MWS at 1-year follow-up. A preoperative risk index including sex, hypertension, ejection fraction (EF) and high hsCRP (index 1) or SAA (index 2) predicted low 1-year MWS with 81% sensitivity/71% specificity (index 1), and 77% sensitivity/77% specificity (index 2) in ROC analyses (AUC 0.80 and 0.79, p < 0.001). Among individuals with severe obesity, women and patients with hypertension, increased serum levels of inflammatory proteins and reduced EF are at high risk of impaired LV midwall mechanics 1 year after bariatric surgery.

**ClinicalTrials.gov identifier** NCT01533142 February 15, 2012.

## Introduction

Obesity tops the burden of metabolic diseases globally and has an alarming annual increase of 3% in adults and above 5% in children^1,2^. Excess adipose tissue impacts every organ system and is associated with increased cardiovascular (CV) risk and subclinical cardiac dysfunction^3–5^. The Global Burden of Disease Study has identified obesity as the direct cause of 11% of heart failure cases in men and 14% in women^6^. In the prospective Bariatric Surgery on the West Coast of Norway study, we have previously found that impaired cardiac mechanics in the longitudinal or radial direction is largely prevalent in middle-aged patients with severe obesity^5^. Despite that, patients with obesity referred to bariatric surgery do not routinely undergo preoperative screening with echocardiography in Norway^7,8^.

Bariatric surgery has established itself as one of the most effective methods for achieving large permanent weight loss^9–11^. Over 15% weight loss is estimated as necessary to reduce CV mortality and heart failure^9–11^. However, despite significantly lower body mass index (BMI) and improvement in CV risk profile and hemodynamics, 44% of patients that undergo bariatric surgery remain with abnormal cardiac function one year after surgery, most often due to reduced left ventricular (LV) midwall mechanics^12^.

The causes of persistent cardiac dysfunction after bariatric surgery are not well understood. Obesity is known to be associated with low-grade chronic inflammation, and in recent years several circulating inflammatory proteins have been revealed as both potentiators of inflammation and mediators of obesity-associated endothelial dysfunction and insulin resistance^13^. High-sensitivity C-reactive protein (hsCRP) and serum amyloid A (SAA) are persistently elevated in obesity and diabetes, and both have been linked to increased mortality in patients with coronary artery disease^14–18^. Both circulating calprotectin and its gene expression in visceral adipose tissue are increased in adults and children with obesity, among whom it is related to higher recruitment of macrophages in the adipose tissue and increased inflammation^19^. Whether established or more novel inflammatory markers can predict LV dysfunction following bariatric surgery has, to our knowledge, not been explored previously.

To better characterize the impact of bariatric surgery on LV cardiac mechanics, we analyzed data from the Bariatric Surgery on the West Coast of Norway study to determine how preoperative inflammation, findings at the initial electrocardiography (ECG) and echocardiography and traditional CV risk factors may predict longer-term cardiac impairment. Based on the identified predictors, we sought to develop a simplified risk index to be used in the preoperative assessment of patients with severe obesity.

## Methods

### Study design

The Bariatric Surgery on the West Coast of Norway study is a prospective follow-up of patients with obesity referred for Roux-en-Y gastric bypass surgery on the West Coast of Norway (2012–2016). The study protocol has been previously reported in detail^5,12,20^. Briefly, the Bariatric Surgery on the West Coast of Norway study enrolled individuals aged 18–60 years eligible for bariatric surgery based on national recommendations (BMI ≥ 40 kg/m^2^ or BMI ≥ 35 kg/m^2^ with at least one weight related condition as hypertension, diabetes mellitus type 2 and/or sleep apnea). Exclusion criterion was pregnancy during the study period. Patients underwent 12-lead ECG and echocardiography, and blood samples were collected at baseline, 6 months, and 1-, 2- and 5-years post-surgery.

A total of 123 patients were recruited in the cardiac study arm. Of these, two patients had known coronary artery disease and were consequently excluded from the analyses. The remaining participants had no history of CV disease at study inclusion. Biobank samples for analysis of inflammatory markers were available in 76 patients preoperatively. Of these, 75 patients had complete echocardiographic examinations both before and 1 year after surgery and thus constituted the present study population. Patients excluded from the present analysis were slightly younger (37 ± 12 vs 44 ± 10 years, p < 0.001), but had similar prevalence of hypertension and diabetes, and comparable sex distribution, BMI and LV mechanics by midwall shortening (MWS) and global longitudinal strain (GLS) with the included patients. Preoperative hypertension was defined as history of hypertension, blood pressure (BP) ≥ 140 mmHg systolic or ≥ 90 mmHg diastolic, or use of antihypertensive drugs. Diabetes was defined as glycated hemoglobin (HbA_1c_) ≥ 6.5%, fasting blood sugar ≥ 7.0 mmol/l, prior diagnosis of diabetes, or use of antidiabetic medications^21^.

The study was approved by the Regional Comittee for Medical Research Ethics Western Norway (2021/22196) and adhered to the revised Declaration of Helsinki. Patients provided written informed consent before enrollment.

### Biobank analyses

Blood samples collected at all study visits were processed as per standard procedures and serum stored at − 80 °C in the West-Norwegian Biobank for Overweight at Haukeland University Hospital before being analyzed at the Bevital laboratory in Bergen, Norway (www.bevital.no). Serum concentrations of the inflammation markers hsCRP, total SAA and amyloid A isoforms (SAA1.1-1.3, SAA2.1-2.2), as well as total calprotectin and calprotectin subunits (S100A8 and S100A9) were analyzed. Cystatin C, a marker of glomerular filtration rate independent of age, sex, muscle mass and diet, was measured as an indicator of renal function. HbA_1c_ was measured as a marker of glycemic control. All inflammatory proteins, cystatin C and HbA_1c_ were measured by matrix-assisted laser desorption/ionization-mass spectrometry (MALDI-TOF MS) approaches^22^. Within- and between-day coefficient of variation for the inflammatory proteins, cystatin C and HbA_1c_ ranged from 4 to 7% and 4–10%, respectively.

### ECG measurements

On preoperative ECGs, atrial depolarization was assessed by the P wave axis and duration. Ventricular de- and repolarization were evaluated by the QRS axis and duration, T axis, QRS-T angle, time to intrinsicoid deflection, and corrected QT time.

### Echocardiographic measurements

The echocardiographic protocol has been previously described^5,12,20^. All images were analyzed by a junior (LMDG) and proofread by a senior researcher (DC) at the Bergen echocardiography core laboratory at University of Bergen.

#### LV structure

LV dimensions and mass were assessed following the joint American Society of Echocardiography and European Association of Cardiovascular Imaging guidelines for quantitative echocardiography^23^. LV hypertrophy was defined as LV mass indexed for height^2.7^ ≥ 49.2 g/m^2.7^ in men and ≥ 46.7 g/m^2.7^ in women^5^. The ratio of LV end-diastolic posterior wall thickness/internal diameter (the relative wall thickness) indicated concentric LV geometry when above 0.42^23^. Pericardial fat was measured in the parasternal long-axis view at end-diastole as previously reported^20^.

#### Left ventricular function and mechanics

LV systolic function was measured by Simpson´s biplane ejection fraction (EF, low if < 52% in men and < 54% in women)^23^. LV wall mechanics was measured in the radial direction by the MWS (low if < 14% in men and < 16% in women). MWS reflects the LV fractional shortening at the level of the midwall and was derived from the two-dimensional LV end-diastolic and end-systolic chamber dimensions and wall thicknesses using a previously prognostically validated formula^24^. Patients with normal/low MWS at both the preoperative and 1-year postoperative visits were classified as having persistently normal or persistently low MWS, respectively. LV mechanics in the longitudinal direction was assessed by the GLS (low if less negative than − 16.7% in men and -17.8% in women)^25^. GLS was analyzed by using all three standard apical views in a vendor-independent software package (2D Cardiac Performance Analysis, Image Arena, Tomtec)^25^. LV pump performance was assessed by the biplane stroke volume. LV myocardial oxygen (O_2_) demand was estimated from the LV mass-wall stress-heart rate product and considered high if > 2.29 × 10^6^ g kdyne/cm^2^ bpm in men, and > 1.62 × 10^6^ g kdyne/cm^2^ bpm in women as previously reported^5,26^.

### Statistical analyses

Statistical analyses were performed in IBM SPSS Statistics 28.0 (IBM Corp., Armonk, NY, USA) as well as R (R core team, 2023, R studio, Posit Team, 2023 and the ggplot2 package, Wickham, 2016). Patients were grouped according to normal vs. low MWS at baseline and 1 year postoperatively.

Normality was evaluated with both Shapiro–Wilk and Q–Q Plots. For normally distributed variables, independent-samples and paired-samples t tests, and one-way ANOVA with Turkey–Kramer post hoc test were used for comparisons between groups. When data was not normally distributed, correlations were assessed by the Spearman test, and differences between groups by bootstrap t tests as appropriate. Categorical variables were compared by the chi-square test. Findings are reported as mean ± standard deviation (SD) or as percentages.

The relation between clinical characteristics, preoperative inflammatory proteins and echocardiographic findings, and low 1-year MWS was assessed initially using univariable regression analyses. HsCRP and serum calprotectin S100A9 were categorized as high if in the highest tertile, and SAA as high if in the highest quartile. Clinically relevant variables and variables associated with low MWS at a 2-sided probability < 0.1 were then entered into multivariable logistic regression analyses run with a combination stepwise selection method. All multivariable models were evaluated by the Hosmer–Lemeshow goodness-of-fit test and the Nagelkerke R^2^ value, and the two best fitted models were further used to construct two risk indexes. The risk indexes included the identified independent predictors of low 1-year MWS in the two final multivariable logistic models, with the relative weight of each predictor calculated from the parameter estimate (B) in the logistic model, multiplied by 10. The risk indexes were then evaluated in terms of predictive ability using the areas under the Receiver Operating Characteristic (ROC) curves (AUC) with 95% confidence intervals (CI) and p values. In both ROC analyses, the value of each risk index with best sensitivity and specificity in predicting low 1-year MWS was identified. A two-tailed p < 0.05 was considered significant in all analyses.

## Results

### Preoperative LV function and inflammation

Before bariatric surgery, 45% (34/75) of patients had low MWS. Despite comparable age, BMI and LV EF, the low preoperative MWS group had higher heart rate and systolic blood pressure as well as higher HbA_1c_ and serum concentration of inflammatory proteins, particularly hsCRP, total calprotectin, the calprotectin subunit S100A9, and SAA isoforms SAA1.3 and SAA2.2 (Table 1). On ECG, patients with low MWS had comparable P wave (97 vs 94 msek) and PR duration (166 vs 159 msek) (p = 0.09) and similar measures of ventricular de- and repolarization. Myocardial O_2_ demand was high in 33% of the study population before surgery and correlated with increased serum calprotectin S100A9 (r = 0.32, p < 0.01). Patients with low preoperative MWS also had smaller LV dimensions with higher relative wall thickness and myocardial O_2_ demand (Table 2).

**Table 1:** Clinical and biochemical characteristics of the whole study population and in patients with normal vs. low LV MWS preoperatively and 1 year after bariatric surgery. The serum concentration of inflammatory proteins is presented for hsCRP, total calprotectin and calprotectin subunits S100A8 and S100A9, as well as for total SAA and for SAA isoforms that were significantly different between patients with normal vs. low MWS (SAA1.1, SAA1.3, SAA2.1, SAA2.2).

	Baseline				1-year follow-up			
	Total cohort (n = 75)	Normal MWS (n = 41)	Low MWS (n = 34)	P value	Total cohort (n = 75)	Normal MWS (n = 49)	Low MWS (n = 26)	P value
Preop age (years)	44 ± 10	43 ± 9	46 ± 10	0.09		43 ± 9	46 ± 10	0.20
Women	72%	68%	77%	0.43		67%	81%	0.22
Weight (kg)	120 ± 20	119 ± 22	121 ± 17	0.63	84 ± 16*	83 ± 13	85 ± 22	0.17
BMI (kg/m^2^)	41.5 ± 4.7	41.0 ± 4.8	42.1 ± 4.6	0.33	28.9 ± 4.7*	28.7 ± 4.0	29.5 ± 5.9	0.06
Heart rate (bpm)	74 ± 13	71 ± 12	78 ± 13	0.03	66 ± 10*	64 ± 7	69 ± 12	0.001
Systolic BP (mmHg)	135 ± 9	133 ± 8	137 ± 11	0.05	127 ± 9*	125 ± 8	130 ± 11	0.22
Diastolic BP (mmHg)	87 ± 7	86 ± 6	88 ± 8	0.15	83 ± 5*	82 ± 5	84 ± 7	0.42
Preop hypertension	56%	46%	68%	0.06		47%	73%	0.03
Preop diabetes	19%	12%	27%	0.11		22%	12%	0.36
Medication								
Antihypertensive	32%	31%	35%	0.72	19%^†^	23%	12%	0.26
Lipid-lowering	16%	18%	12%	0.44	6%^†^	8%	0%	na
Antidiabetic	17%	22%	8%	0.11	8%^†^	10%	4%	0.34
HbA_1c_ (%)	5.90 ± 1.23	5.59 ± 1.04	6.26 ± 1.35	0.03^§^	5.40 ± 0.77*	5.40 ± 0.72	5.40 ± 0.87	0.94§
Total cystatin C (µg/ml)	1.11 ± 0.24	1.08 ± 0.25	1.16 ± 0.23	0.12^§^	1.08 ± 0.22	1.10 ± 0.21	1.05 ± 0.22	0.48§
hsCRP (µg/ml)	5.68 ± 4.12	4.48 ± 3.21	7.13 ± 4.51	0.005^§^	1.66 ± 1.81*	1.22 ± 1.06	2.51 ± 2.56	0.01§
Calprotectin S100A8 (µg/ml)	1.15 ± 0.59	1.04 ± 0.56	1.27 ± 0.60	0.08^§^	0.84 ± 0.44*	0.83 ± 0.41	0.86 ± 0.51	0.81§
Calprotectin S100A9 (µg/ml)	0.40 ± 0.19	0.35 ± 0.18	0.46 ± 0.19	0.02^§^	0.34 ± 0.19	0.35 ± 0.20	0.34 ± 0.18	0.85§
Total calprotectin (ug/ml)	2.03 ± 0.92	1.83 ± 0.86	2.27 ± 0.93	0.04^§^	1.64 ± 0.77*	1.64 ± 0.75	1.64 ± 0.82	0.99§
SAA1.1 (µg/ml)	1.10 ± 1.70	0.85 ± 1.19	1.40 ± 2.14	0.09^§^	0.50 ± 0.95^†^	0.30 ± 0.30	0.90 ± 1.53	0.02§
SAA1.3 (µg/ml)	0.27 ± 0.41	0.19 ± 0.28	0.37 ± 0.51	0.03^§^	0.13 ± 0.33*	0.07 ± 0.11	0.26 ± 0.52	0.03§
SAA2.1 (µg/ml)	0.42 ± 0.71	0.35 ± 0.69	0.50 ± 0.74	0.40^§^	0.18 ± 0.31*	0.11 ± 0.14	0.30 ± 0.48	0.02§
SAA2.2 (µg/ml)	0.17 ± 0.32	0.11 ± 0.23	0.24 ± 0.41	0.05^§^	0.09 ± 0.18*	0.05 ± 0.08	0.16 ± 0.28	0.04§
Total SAA (µg/ml)	3.79 ± 5.29	3.17 ± 4.67	4.55 ± 5.94	0.23^§^	1.75 ± 2.95^†^	1.05 ± 1.08	3.12 ± 4.61	0.01§

*BMI* body mass index, *BP* blood pressure, *HbA*_*1c*_ glycated hemoglobin, *hsCRP* high-sensitivity C-reactive protein, *MWS* midwall shortening, *na* not applicable, *SAA* serum amyloid A.

*P value*s in the last column indicate the level of significance when comparing patients with normal vs. low MWS preoperatively or 1 year after surgery and are based on independent-samples t tests for normally distributed variables, and bootstrap t tests (indicated by ^§^) for not-normally distributed variables. *p < 0.001 and ^†^*p* < 0.01 indicate the level of significance when comparing preoperative and postoperative values in the whole population by paired-samples t tests for normally distributed variables and by paired bootstrap t tests for not-normally distributed variables.

**Table 2:** Echocardiographic characteristics of the whole study population and in patients with normal vs. low LV MWS preoperatively and 1 year after bariatric surgery.

	Baseline				1-year follow-up			
	Total cohort (n = 75)	Normal MWS (n = 41)	Low MWS (n = 34)	P value	Total cohort (n = 75)	Normal MWS (n = 49)	Low MWS (n = 26)	P value
LV end-diastolic diameter (cm)	5.11 ± 0.40	5.20 ± 0.34	5.00 ± 0.44	0.03	4.95 ± 0.43*	5.05 ± 0.38	4.77 ± 0.46	0.007
LV end-systolic diameter (cm)	3.43 ± 0.39	3.37 ± 0.39	3.52 ± 0.38	0.10	3.35 ± 0.34	3.31 ± 0.28	3.44 ± 0.44	0.12
LV end-diastolic volume (mL)	120 ± 32	122 ± 32	117 ± 31	0.43	135 ± 30*	137 ± 30	131 ± 31	0.39
LV mass index (g/m^2.7^)	46 ± 13	43 ± 10	49 ± 15	0.02^§^	40 ± 11*	39 ± 11	40 ± 12	0.72^*§*^
LV hypertrophy	40%	32%	50%	0.11	21%*	18%	27%	0.40
Relative wall thickness	0.33 ± 0.06	0.30 ± 0.05	0.36 ± 0.07	< 0.001^§^	0.34 ± 0.07	0.32 ± 0.05	0.38 ± 0.08	< 0.001^§^
Pericardial fat (mm)	12.7 ± 3.9	12.0 ± 3.7	13.5 ± 4.1	0.10^§^	10.2 ± 3.1*	9.9 ± 2.9	10.5 ± 3.3	0.42^§^
LV EF (%)	61 ± 6	61 ± 6	61 ± 6	0.72	60 ± 6	60 ± 6	59 ± 5	0.33
Low EF	8%	7%	9%	1.00	10%	4%	21%	0.02
Stroke volume (mL)	99 ± 30	99 ± 23	98 ± 38	0.90^§^	101 ± 20	103 ± 19	96 ± 22	0.17^§^
MWS (%)	16.0 ± 2.6	17.7 ± 2.1	13.9 ± 1.4	na	16.2 ± 2.6	17.5 ± 2.0	13.6 ± 1.5	na
GLS (%)	− 15.6 ± 4.9	− 15.7 ± 4.9	− 15.6 ± 4.8	0.95	− 20.2 ± 2.5*	− 20.6 ± 2.6	− 19.4 ± 2.2	0.05
Low GLS	61%	58%	63%	0.67	13%*	14%	13%	0.84
LV mass-wall stress-heart rate product (× 10^6^ g kdyne/cm^2^ bpm)	1.68 ± 0.60	1.49 ± 0.57	1.90 ± 0.57	0.004§	1.16 ± 0.42*	1.06 ± 0.32	1.34 ± 0.53	0.007^§^
High LV mass-wall stress-heart rate product	33%	24%	44%	0.071	7%*	2%	15%	0.03

*EF* ejection fraction, *GLS* global longitudinal strain, *LV* left ventricle, *MWS* midwall shortening, *na* not applicable.

*P value*s in the last column indicate the level of significance when comparing patients with normal vs. low MWS preoperatively or 1 year after surgery and are based on independent-samples t tests for normally distributed variables, and bootstrap t tests (indicated by ^§^) for not-normally distributed variables. *p < 0.001 indicates the level of significance when comparing preoperative and postoperative values in the whole population by paired-samples t tests for normally distributed variables and by paired bootstrap t tests for not-normally distributed variables.

### Postoperative LV function and inflammation

The mean follow-up was 14 ± 3 months. Bariatric surgery induced a significant fall in serum hsCRP, total serum calprotectin and total SAA, as well as an improvement in both GLS and myocardial O_2_ demand (all p < 0.01) (Tables 1 and 2). High myocardial O_2_ demand was present in 7% of patients 1 year after surgery (Table 2), and in this group the preoperative serum levels of all inflammatory proteins were significantly higher than in patients with normalized O_2_ demand after surgery (all p < 0.05).

**Figure 1:**
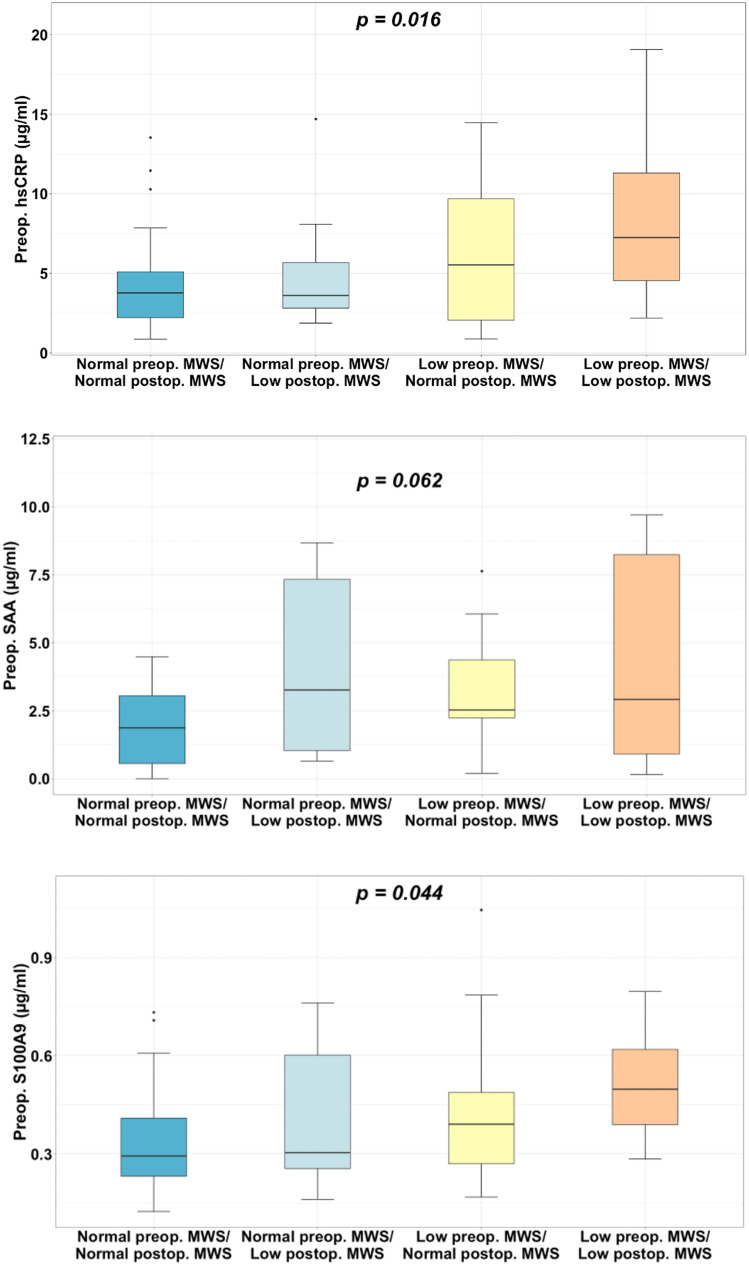
Preoperative serum concentration of inflammatory proteins in patients with persistently normal MWS, normal preoperative MWS and low postoperative MWS, low preoperative MWS that normalized postoperatively, and persistently low MWS. *p-values are based on one-way ANOVA.

**Figure 2:**
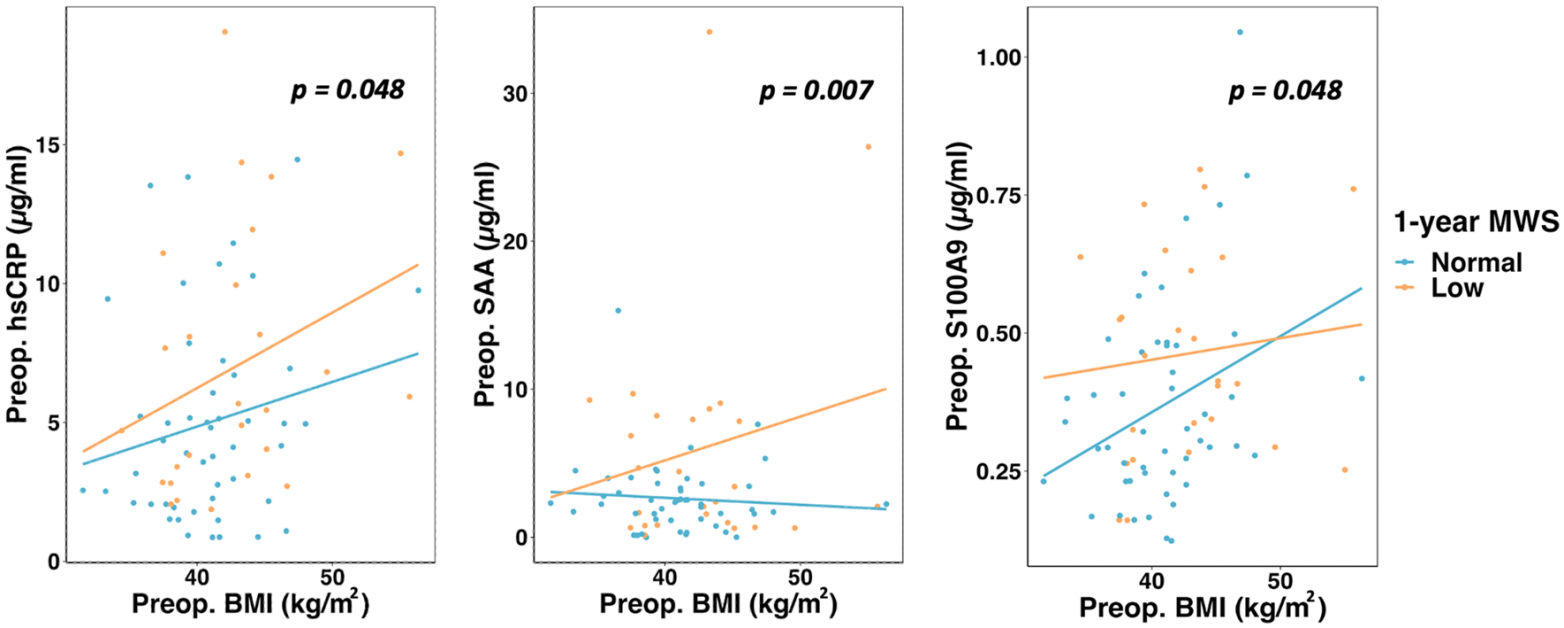
Preoperative serum concentration of inflammatory proteins according to preoperative BMI in patients with low vs. normal 1-year postoperative MWS. *p-values are based on bootstrap t-test.

MWS remained on average unchanged (Table 2), and 35% of patients had low MWS 1 year after surgery. Of these, 62% also presented with low MWS before surgery, i.e. persistently low MWS. Patients with low 1-year LV midwall function had higher heart rate, more often hypertension and higher levels of both hsCRP, calprotectin S100A9 and total SAA preoperatively (all p < 0.05). When analyzing the preoperative serum concentration of inflammatory markers in relation to changes in MWS after surgery, the level of inflammatory proteins (in particular hs-CRP and calprotectin subunit S100A9) was highest in patients with persistently low MWS, and lowest in those with persistently normal MWS (Fig. 1). Patients with low 1-year MWS had significantly higher preoperative concentrations of both hsCRP and SAA across the BMI range, and of calprotectin subunit S100A9 in patients with BMI up to 50 kg/m^2^, compared to patients with normal 1-year postoperative MWS (Fig. 2).

When preoperative echocardiographic findings were compared, patients with low 1-year MWS had lower EF: 59 ± 7% vs. 62 ± 5% and significantly higher myocardial oxygen demand before surgery: 1.85 ± 0.69 vs 1.58 ± 0.53 g kdyne/cm^2^ bpm × 10^6^ (both p < 0.05). Preoperative ECG measures of atrial and ventricular function did not differ between patients with normal and low MWS 1 year after surgery. Postoperatively, patients with low MWS had higher relative wall thickness, lower GLS and higher myocardial oxygen demand (Table 2).

### Sex and inflammation in severe obesity and after bariatric surgery

Women were numerically overrepresented among patients with low MWS both pre- and post-operatively (Table 1) despite having similar age (44 ± 10 years in both sexes) and preoperative BMI (41.2 ± 4.5 kg/m^2^ in women vs. 42.4 ± 5.2 kg/m^2^ in men, p = 0.32), and lower preoperative BP than men (systolic BP 133 ± 9 mmHg in women vs. 140 ± 8 mmHg in men; diastolic BP 86 ± 7 mmHg in women vs. 91 ± 6 mmHg in men, both p < 0.01). Twenty-five percent of women vs. 10% of men had persistently low MWS 1 year after surgery. At baseline, the concentration of inflammatory proteins did not differ between sexes (Table 3). However, when comparing patients with preoperative BMI below or above the median (41.2 kg/m^2^), hsCRP was highest in women with BMI above the median (p < 0.05, Supplemental figure). Surgery resulted in a reduction of hsCRP and SAA in both sexes (Table 3). Compared to patients with persistently normal MWS, women with persistently low MWS had higher preoperative serum calprotectin S100A9, and men with persistently low MWS had higher preoperative serum SAA (both p < 0.05).

**Table 3:** Comparison between circulating inflammatory proteins in women and men preoperatively and 1 year after bariatric surgery. The serum concentration of inflammatory proteins is presented for hsCRP, total calprotectin and calprotectin subunits S100A8 and S100A9, as well as for total SAA.

	Women (n = 54)			Men (n = 21)		
	Baseline	1-year follow-up	P value	Baseline	1-year follow-up	P value
hsCRP (µg/ml)	5.87 ± 4.00	1.81 ± 1.95	< 0.001	5.18 ± 4.53	1.31 ± 1.40	< 0.001
Calprotectin S100A8 (µg/ml)	1.13 ± 0.58	0.89 ± 0.47	0.01	1.18 ± 0.62	0.71 ± 0.35	< 0.001
Calprotectin S100A9 (µg/ml)	0.41 ± 0.19	0.35 ± 0.19	0.32	0.39 ± 0.19	0.34 ± 0.22	0.19
Total calprotectin (µg/ml)	2.02 ± 0.91	1.70 ± 0.77	0.07	2.04 ± 0.94	1.50 ± 0.75	0.004
Total SAA (µg/ml)	3.28 ± 3.06	1.80 ± 2.90	0.04	5.10 ± 8.73	1.65 ± 3.17	0.03

*hsCRP* high-sensitivity C-reactive protein, *SAA* serum amyloid A.

*P value*s in the last column indicate the level of significance when comparing baseline vs. 1-year values in women and separately in men by paired bootstrap t tests.

### Risk index for postoperative LV dysfunction

**Figure 3:**
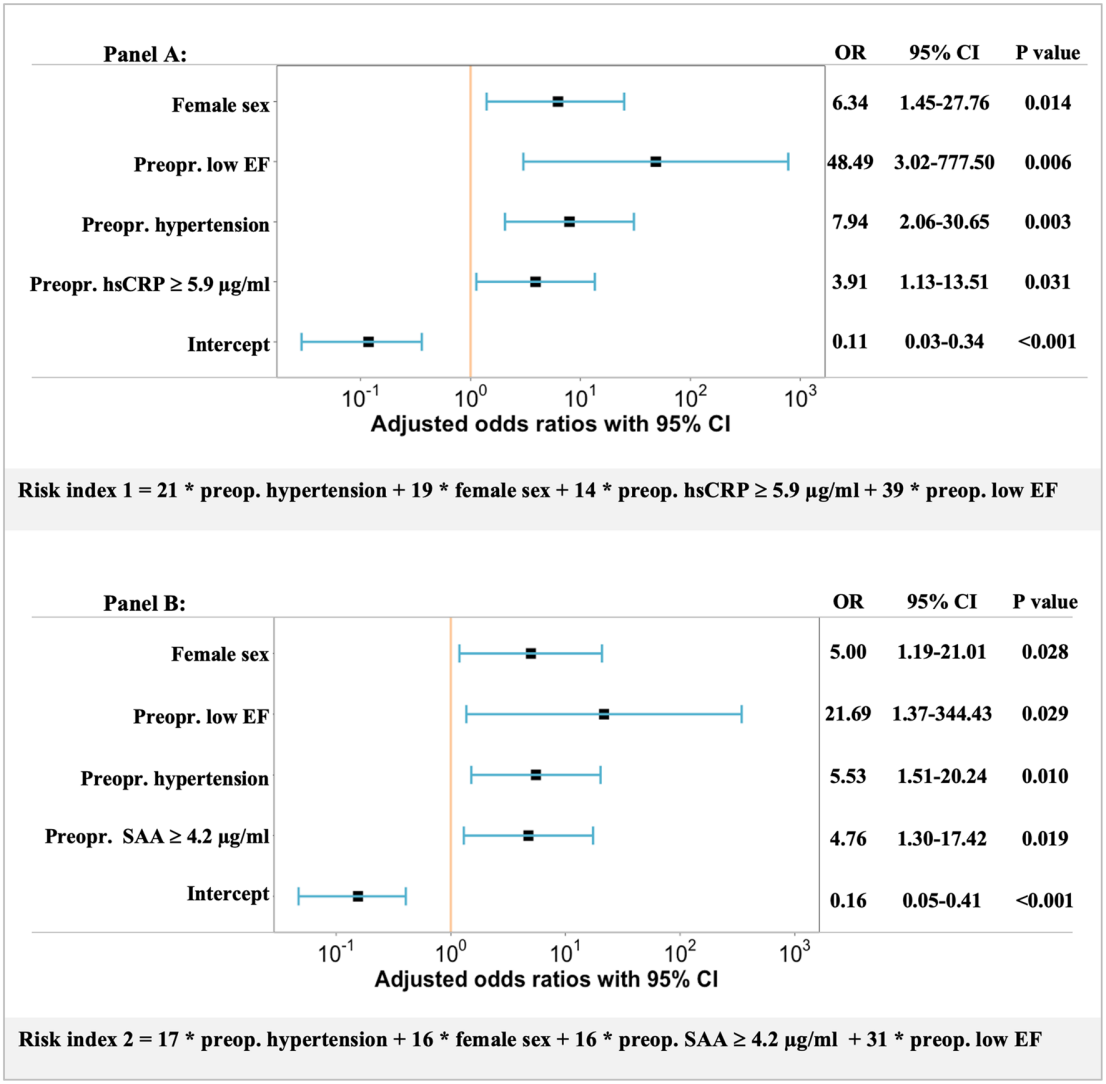
Forests plots showing the odds ratios (OR) with 95% CIs for the variables associated with low 1-year MWS in two logistic regression models (**A** and **B**). The risk score built on each of the logistic regression model is presented under the respective panel.

In logistic regression analysis, low LV MWS 1 year after surgery was significantly associated with the following preoperative features: hypertension, female sex, low LV EF and hsCRP in the highest tertile (above 5.9 µg/ml) (− 2 Log likelihood 72.2, Nagelkerke R^2^ 0.38, p = 0.01 for the overall model) (Fig. 3).

In a similar regression analysis, substituting high hsCRP with high SAA (i.e. above 4.2 µg/ml), SAA was also independently related to low 1-year MWS (− 2 Log likelihood 71.4, Nagelkerke R^2^ 0.39, p = 0.01 for the overall model, Fig. 3).

In a third regression model, replacing hsCRP and SAA with serum calprotectin S1009, the latter was not statistically significant (p = 0.07). In subsequent analyses, age, preoperative BMI or diabetes, surgery-induced reduction in BMI, as well as use of antihypertensive, antidiabetic and cholesterol lowering drug treatment did not independently predict low 1-year MWS.

**Figure 4:**
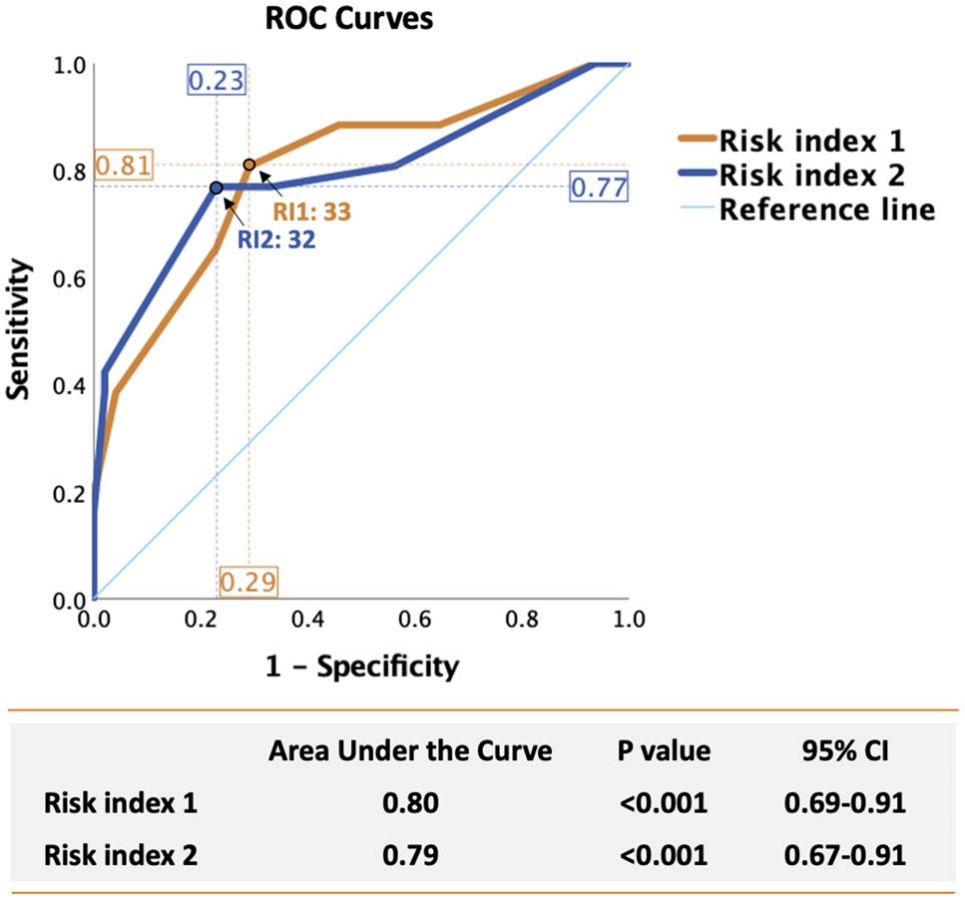
Receiver Operating Characteristic analyses for the association between preoperative risk index 1 (hsCRP-based, orange line) and risk index 2 (SAA-based, blue line) and low 1-year postoperative LV MWS. The discriminating power of each risk index is indicated by the area under curve (AUC) with 95% CI. Cut-off values with best sensitivity and 1-specificity are indicated for each risk core.

Two risk indexes for low 1-year MWS were developed based on the identified preoperative predictors: sex, hypertension, EF and elevated hsCRP (risk index 1) or elevated SAA (risk index 2) (Fig. 3). In ROC analyses, both models had good discriminating power with AUC of 0.80 and 0.79, respectively (Fig. 4). A risk index 1 above 33 identified patients with low 1-year MWS with 81% sensitivity and 71% specificity, while a risk index 2 above 32 distinguished patients with low 1-year MWS with 77% sensitivity and 77% specificity (Fig. 4).

## Discussion

Middle-aged patients with severe obesity and without known cardiac disease have an unfavorable CV risk profile and often abnormal cardiac mechanics. In 44% of these patients, LV mechanics remains low 1 year after bariatric surgery despite large weight loss^12^. Contrary to this evidence, multiple national guidelines for screening of patients with severe obesity referred to bariatric surgery, including the Norwegian guidelines, do not recommend a preoperative cardiological examination. In this subanalysis of the Bariatric Surgery on the West Coast of Norway study, we analyzed factors associated with impaired LV mechanics after surgery using combined preoperative assessment of comorbidities, routine echocardiography and inflammatory serum proteins. Our results pointed out women with obesity, hypertension, higher levels of chronic inflammation (particularly increased hsCRP or SAA) and impaired preoperative LV EF as the patients at the highest postoperative risk, suggesting that this group needs stricter CV risk control and may benefit from integrated cardiological follow-up after surgery.

### Inflammatory proteins and cardiac function in severe obesity

Obesity is characterized by chronic low-grade inflammation^19,27^. The large adipose tissue volume present in individuals with severe obesity creates a pro-inflammatory milieu as a consequence of macrophage and T-cell infiltration, accumulation of pro-inflammatory cytokines and ultimately insulin resistance and dysregulation of glucose and lipid metabolism^28^. However, the level of inflammation varies between people with obesity. A metabolically healthy obesity phenotype, characterized by low levels of serum inflammatory markers has been previously described^29^. In patients that are metabolically “unhealthy” with higher levels of circulating inflammatory proteins, it has been uncertain whether inflammation contributes to impaired cardiac function.

We have earlier shown that half of middle-aged patients with severe obesity and no clinical cardiac disease have impaired cardiac mechanics as indicated by MWS^12^. MWS has been proven to be prognostically important in different cardiac overload conditions and is depressed in systemic inflammatory diseases such as hypertension and rheumatoid arthritis^30^. The association between levels of circulating inflammatory proteins and impaired MWS however has not been thoroughly investigated. One previous report from Taiwan found in a retrospective analysis of 1071 subjects recruited from the general population an association between mildly increased hsCRP and low LV MWS in women^31^. In our cohort with severe obesity, both well-established (hsCRP and total calprotectin) as well as more novel inflammatory proteins (including calprotectin subunit S100A9 and several amyloid isoforms) were significantly higher in serum from patients with low midwall mechanics, a group with comparable BMI and EF, but higher clustering of traditional CV risk factors (including hypertension and diabetes) compared to patients with preserved LV MWS. Interestingly, hsCRP was highest in women with the most severe degree of obesity, and this group also had the highest preoperative prevalence of low MWS. These findings link inflammatory proteomics to impaired midwall cardiac mechanics in individuals with a metabolically “unhealthy” obesity phenotype.

### At risk preoperative phenotype and postoperative cardiac dysfunction

Bariatric surgery improves CV risk profile and outcomes^32^. However, it does not normalize cardiac mechanics in all patients, particularly not in the radial direction. After stepwise testing of multiple risk factors, we have identified female sex, hypertension, low preoperative EF and increased preoperative serum hsCRP or SAA as strongly associated with higher risk of LV dysfunction 1 year after bariatric surgery. Low EF was chosen due to its predictive value and because it is part of every standard echocardiography, including focused protocols. Integration of these factors in a risk index discriminated well between patients with normal and impaired function postoperatively. Of note, a standard preoperative echocardiography was more prognostically important in our patients than screening with ECG alone, as no preoperative ECG marker of atrial or ventricular activity predicted postoperative cardiac dysfunction.

Changes in cardiac mechanics after bariatric surgery have not been previously studied in detail in relation to the preoperative level of inflammation. We demonstrate that patients with low 1-year LV MWS had higher baseline concentration of hsCRP and SAA at all BMI levels. Of note, a gradient was observed in the preoperative levels of hsCRP and calprotectin unit S100A9, from patients with persistently normal MWS (lowest levels) to patients with persistently impaired MWS (highest levels), confirming the consistency of the relationship between chronic inflammation and low midwall mechanics. In experimental models, increased SAA has been shown to stimulate cardiac fibrosis by increasing the effects of transforming growth factor β on cardiac fibroblasts^33^. In a substudy from MESA (Multi-Ethnic Study of Atherosclerosis) on 772 participants free of CV disease and with an average BMI of 28 kg/m^2^, serum hsCRP as well as the pro-inflammatory cytokine interleukin 6 were both associated with increased interstitial myocardial fibrosis by cardiac magnetic resonance in men^34^. Taken together, these studies suggest a possible mechanistic link between chronic inflammation in severe obesity, adverse myocardial structural remodeling, and impaired midwall mechanics after bariatric surgery.

Traditional CV risk factors and in particular hypertension before bariatric surgery predicted a high risk of low 1-year MWS in our population after multiple adjustments. Several experimental and clinical studies have pointed out the role of chronic inflammation in the development and progression of hypertension, and concomitant hypertension and increased BMI have also earlier been shown to be detrimental for LV midwall function^35,36^. Low EF was present in a low proportion of patients preoperatively, but was the predictor strongest related to low postoperative midwall mechanics, reflecting a more advanced stage of myocardial disease.

Despite earlier evidence that women have a better preserved midwall function in chronic overload conditions such as hypertension^37^ and aortic stenosis, our study found that women with severe obesity more frequently experienced impaired MWS 1 year after bariatric surgery. It is well documented that the distribution of adipose tissue is sex-specific, with women presenting more subcutaneous and less visceral fat than men. The subcutaneous adipose tissue volume has very recently been shown to be associated with the level of circulating leukocytes and many inflammatory proteins in women, but not in men living with obesity^38^. This challenges the concept of subcutaneous adipose tissue as benign and shows that it can induce systemic inflammation and further cardiometabolic complications, particularly in women. A cardiac magnetic resonance study found an association between the amount of pericardial fat surrounding the left ventricle and cardiac function in women with obesity, suggesting a local detrimental effect of pericardial fat on the LV myocardium that might be sex-specific^39^. The expression of calprotectin subunit S100A9 is increased in people with obesity in whom it promotes inflammation in the epicardial fat^40^. In mice, overexpression of S100A9 in the cardiomyocytes is linked to reduced calcium flux and depressed LV EF^41^. In our study, preoperative serum S100A9 was overall not independently associated with impaired LV mechanics after surgery, but had higher values in women with persistently low vs persistently normal 1-year MWS. Further research on the mechanisms behind sex-specific changes in cardiac mechanics after bariatric surgery is needed.

### Clinical implications

Impaired LV function is common in patients undergoing bariatric surgery for severe obesity. Understanding the consequences of bariatric surgery on cardiac mechanics and identifying the patients who require closer postoperative follow-up is therefore highly clinically relevant. We propose a simple, pragmatic risk score composed of four variables as an easy-to-use tool in the preoperative screening of these patients. The risk index requires clinical profiling, measurement of one inflammatory protein and a focused echocardiography with assessment of LV EF. We encourage referral to a preoperative cardiac examination, stricter risk factors control and cardiological follow-up of patients with cardiac dysfunction, in particular women with obesity, hypertension and higher levels of inflammatory markers.

### Study limitations

The proposed risk index has been constructed based on findings in the Bariatric Surgery on the West Coast of Norway population and should be further validated in an external cohort. Serum samples for analysis of inflammatory proteins were available in 76 out of the 123 patients included in the study. Still novel and significant associations between inflammatory proteomics and impaired LV mechanics was demonstrated. More women than men were recruited in the Bariatric Surgery on the West Coast of Norway study, similar to the sex distribution seen in other bariatric surgery cohorts. Statistically, this might have influenced the ability to detect sex differences in some inflammatory protein isoforms. However, the study was sufficiently powered to identify female sex as a significant prognosticator of 1-year postoperative cardiac dysfunction and consequently sex was included in the proposed risk index.

## Conclusion

One-third of patients with severe obesity and in particular women with hypertension, higher level of inflammation and reduced EF, have impaired LV midwall mechanics 1 year after bariatric surgery. Preoperative assessment by combined echocardiography and measurement of circulating inflammatory proteins may be useful in the routine evaluation of patients referred to bariatric surgery.

### Supplementary Information

Supplementary Figures.

## Data Availability

The data that support the findings of this study are available from the corresponding author upon reasonable request.

## Abbreviations

BMI: Body mass index
BP: Blood pressure
CV: Cardiovascular
ECG: Electrocardiography
EF: Ejection fraction
GLS: Global longitudinal strain
HbA_1c_: Glycated hemoglobin
HsCRP: High-sensitivity C-reactive protein
LV: Left ventricular
MALDI-TOF MS: Matrix-assisted laser desorption/ionization-mass spectrometry
MWS: Midwall shortening
SAA: Serum amyloid A
