# Supplementary material for: Preoperative risk factors associated with left ventricular dysfunction after bariatric surgery
Source: Sci Rep. 2024 Jan 25;14:2173. doi: 10.1038/s41598-024-52623-1 (PMC10810803; doi:10.1038/s41598-024-52623-1)

**Supplemental figure.** Preoperative serum concentration of hsCRP in women and men grouped by the median preoperative BMI of 41.2 kg/m^2^. *P-values are based on bootstrap t-test.


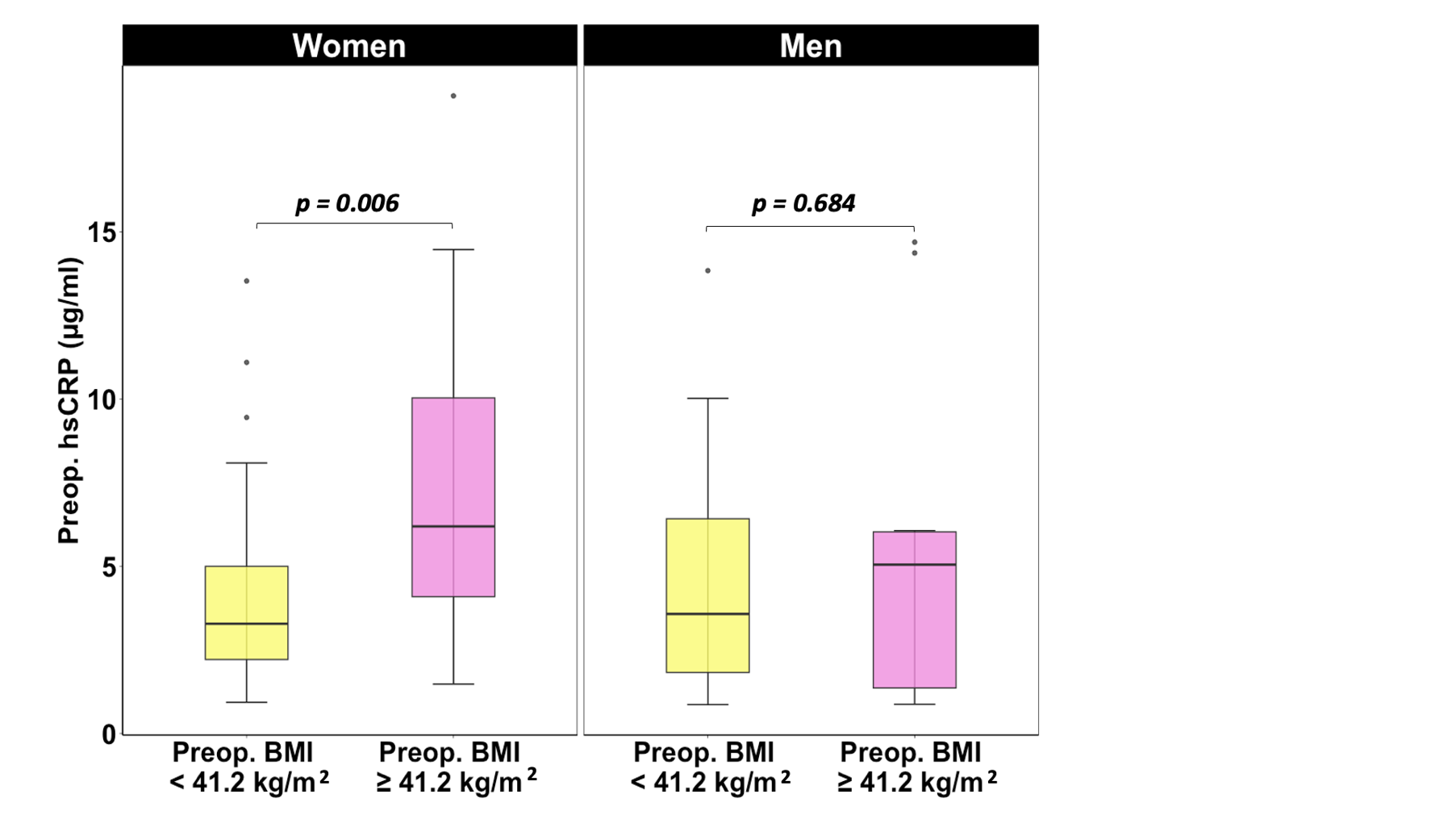

Supplement: Supplementary file 1 — Supplementary Figures. [file 41598_2024_52623_MOESM1_ESM.docx]
